# Changes in Proteins in Saliva and Serum in Equine Gastric Ulcer Syndrome Using a Proteomic Approach

**DOI:** 10.3390/ani12091169

**Published:** 2022-05-02

**Authors:** Alberto Muñoz-Prieto, Maria Dolores Contreras-Aguilar, Jose Joaquín Cerón, Ignacio Ayala, Maria Martin-Cuervo, Juan Carlos Gonzalez-Sanchez, Stine Jacobsen, Josipa Kuleš, Anđelo Beletić, Ivana Rubić, Vladimir Mrljak, Fernando Tecles, Sanni Hansen

**Affiliations:** 1 Clinic for Internal Diseases, Faculty of Veterinary Medicine, University of Zagreb, Heinzelova 55, 10000 Zagreb, Croatia; jkules@vef.unizg.hr (J.K.); abeletic@vef.hr (A.B.); irubic@vef.unizg.hr (I.R.); vmrljak@vef.unizg.hr (V.M.); 2 Interdisciplinary Laboratory of Clinical Analysis of the University of Murcia (Interlab-UMU), Department of Animal Medicine and Surgery, Veterinary School, Regional Campus of International Excellence Mare Nostrum, Campus de Espinardo, University of Murcia, 30100 Murcia, Spain; mariadolores.contreras@um.es (M.D.C.-A.); jjceron@um.es (J.J.C.); ftecles@um.es (F.T.); 3 Department of Animal Medicine & Surgery, Veterinary School, Regional Campus of International Excellence Mare Nostrum, Campus de Espinardo, University of Murcia, 30100 Murcia, Spain; iayape@um.es; 4 Medicine Animal, Faculty of Veterinary Medicine of Cáceres, University of Extremadura, Avenida de la Universidad S-N, 10002 Cáceres, Spain; mmcvet@hotmail.com; 5 BioQuant, Faculty of Biosciences, Heidelberg University, Im Neuenheimer Feld 267, 69117 Heidelberg, Germany; juan-carlos.gonzalez@bioquant.uniheidelberg.de; 6 Department of Veterinary Clinical Sciences, Section Medicine and Surgery, University of Copenhagen, Agrovej 8, 2630 Taastrup, Denmark; stj@sund.ku.dk (S.J.); sannih@sund.ku.dk (S.H.)

**Keywords:** horse, EGUS, proteomics, saliva, serum, EGGD, ESGD

## Abstract

**Simple Summary:**

Equine gastric ulcer syndrome (EGUS) is a highly prevalent disease with a major clinical importance due to its negative effects on the welfare and performance of horses. EGUS can be distinguished into two different diseases depending on which anatomical region is affected: equine glandular gastric disease (EGGD), in which there is a lesion in the glandular stomach, and equine squamous gastric disease (ESGD), in which the alteration appears in the non-glandular stomach. EGUS has nonspecific clinical signs, and its underlying mechanism has not been completely elicited. Therefore, it would be of interest to clarify the pathophysiology and identify potential biomarkers of this syndrome. This study detected differences in the salivary and serum proteome between horses with EGUS and healthy horses, and also between horses with EGGD and ESGD. The most upregulated proteins in EGGD were related to the immune activation whereas, in horses with ESGD, the proteins with the most significant changes were associated with the squamous cell regulation and growth. Compared to serum, saliva had a higher number of proteins showing significant changes and also showed a different pattern of changes, indicating that the proteins in both fluids show a different response to the disease and can provide complementary information.

**Abstract:**

Changes in the salivary proteome in 12 horses with the two diseases included in equine gastric ulcer syndrome (EGUS), equine glandular gastric disease (EGGD) (*n* = 6) and equine squamous gastric disease (ESGD) (*n* = 6), were evaluated using a high-resolution LC-MS/MS analysis of TMT-labelled peptides and compared to 10 healthy control horses. Serum was also analysed for comparative purposes. The comparison between the horses with EGGD and controls showed significant changes in 10 salivary proteins, whereas 36 salivary proteins were differently abundant between ESGD and control groups. The most upregulated proteins in the case of EGGD were related to immune activation whereas, in horses with ESGD, the most significantly changed proteins were associated with squamous cell regulation and growth. Compared to serum, saliva showed a higher number of proteins with significant changes and a different pattern of changes. The proteins identified in our study, in addition to providing new information about the pathophysiological mechanisms in these diseases, could have the potential to be novel biomarkers for the diagnosis or monitoring of EGGD and ESGD.

## 1. Introduction

Equine gastric ulcer syndrome (EGUS) is a highly prevalent disease with a high importance due to its negative effects on the welfare and performance of horses [1,2]. According to the European College of Equine Internal Medicine (ECEIM) Consensus Statement, EGUS can be distinguished into two different diseases, depending on which anatomical region is affected: equine squamous gastric disease (ESGD), in which there is a lesion in the non-glandular stomach; and equine glandular gastric disease (EGGD), in which the alteration appears in the glandular stomach [1,3]. EGUS has nonspecific clinical signs and its mechanism has not been totally elicited. The most common clinical signs that can be observed include poor appetite, loss of weight, yawning, bruxism, anorexia, salivation, abdominal discomfort, and reduced performance [2]. The only antemortem diagnostic method currently validated and considered as a gold standard is the evaluation of the entire stomach using gastroscopy, in order to visualize the specific lesions and their locations [4]. Due to the current lack of specific haematological or biochemical markers to diagnose EGUS, it would be of interest to discover analytes that could potentially serve as biomarkers of this syndrome [2,5,6].

Proteomics allows the identification of a high number of proteins simultaneously, thus being useful in the identification of proteins that can change in a selected disease [7]. Tandem mass tag (TMT) is a labelling procedure that allows the quantification of different peptides, which are marked and identified [8]. This technique has been used previously to investigate the salivary proteome in horses with acute abdominal syndrome [9]. In a previous study, an evaluation of changes in the serum proteins of horses with ESGD using gel electrophoresis and mass spectrometry has been performed [3]. In this report, 10 serum proteins were found to be possible biomarkers for ESGD. However, to the best of the current authors’ knowledge, there are no proteomic studies that evaluate changes in the proteome of saliva in EGUS, and the possible differences between ESGD and EGGD.

The hypothesis of this study is that ESGD and EGGD could produce changes in the proteins in saliva that could be detected by gel-free proteomics. Therefore, the objective was to evaluate the changes produced in saliva proteins of horses with ESGD, EGGD, and healthy horses through proteomic analysis using TMT. Serum was also analysed for comparative purposes. This data can contribute to a better understanding of the pathophysiological changes that occur in these diseases and to identify new potential biomarkers of these conditions.

## 2. Materials and Methods

### 2.1. Animals

Saliva and serum samples from 10 healthy horses and 12 horses with EGUS were included. In the EGUS group, there were 6 horses with each of the different presentation of the disease (EGGD and ESGD). Therefore, the animals included in this study were:

- EGGD horses (*n* = 6; 3 geldings and 3 mares; mean age = 14.6 years (range 5–20); warmblood breeds (*n* = 4) and ponies (*n* = 2)). The presence of EGGD was diagnosed based on gastroscopic examination and the presence of compatible lesions in the glandular mucosa region of the stomach;
- ESGD horses (*n* = 6; 3 geldings and 3 mares; mean age = 11 years (range 5–12); warmblood breeds (*n* = 4), trotter breed (*n* = 1), and Tinker breed (*n* = 1)). All horses had at least a grade 2/4 lesion identified during gastroscopy [1,10] according to the ECEIM Consensus Statement 1).
- Healthy horses (*n* = 10; 7 geldings and 3 mares; mean age = 9.6 years (range 4–22); warmblood breeds (*n* = 3), Spanish pure breed (*n* = 1), ponies (*n* = 2), trotter breed (*n* = 1), Icelandic horse (*n* = 1), and crossbreds (*n* = 2)). These horses were found healthy based on history, clinical examination, complete blood count (CBC), and serum biochemistry profile. In addition, a gastroscopy study was performed to rule out EGUS.

All animals included in this study visited the Large Animal Teaching Hospital at the University of Copenhagen between February 2020 and October 2021. The diseased horses were diagnosed at that hospital by an internist with 10 years of experience from a specialized equine hospital (S.H.).

EGUS was suspected in horses based on compatible symptomatology (weight loss, pain behaviours, changes in temperament or reduced performance) and were referred to the hospital the day before the gastroscopy. Horses fasted for 12 h before the gastroscopy, which was performed as previously described [2]. Images from gastroscopy were used for the EGUS diagnosis [11] and classification according to the ECEIM Consensus Statement [1]. Based on this, horses were stratified into ESGD using the 4-point scale gradation or EGGD. Three of the horses diagnosed with ESGD showed 3 points of severity and three horses showed 2 points of severity.

The healthy horses were privately owned horses with no signs of illness. Gastroscopy was performed to rule out EGUS (using ESGD grading system equal to 0, meaning that epithelium is intact with no hyperkeratotic areas nor any glandular lesions). Horses were included in the healthy population if they had normal results upon physical examination (heart rate, respiratory rate, rectal temperature, colour of mucous membranes, capillary refill time, borborygmi) and they were free of any organ-related pathology based on haematological or biochemical findings.

### 2.2. Sampling of Saliva and Serum Specimens

The saliva samples were collected in all horses before intravenous sedation and gastroscopy, immediately after the horses were placed in the examination stand, as previously reported [12,13]. Blood samples were obtained by jugular venepuncture after the saliva collection. All tubes with saliva and all blood samples were centrifuged at 3000× *g* for 10 min at 4 °C and stored at −80 °C until analysis. All serum samples did not have visual gross haemolysis and all saliva samples did not have evidence of blood contamination according to the colour scale previously reported [14].

### 2.3. Proteomic Analysis

#### 2.3.1. Sample Preparation

Proteomic analysis of saliva and serum samples was performed using the tandem mass tag (TMT)-based quantitative approach as previously described, with minor modifications [15]. Briefly, protein concentration was determined using bicinchoninic acid assay (BCA, Thermo Scientific, Rockford, IL, USA). Afterwards, 35 µg of the samples and internal standards (a pool of equal protein amounts from all samples) were reduced with 200 mM dithiothreitol (DTT, Sigma-Aldrich, St. Louis, MO, USA), alkylated with 375 mM iodoacetamide (Sigma-Aldrich, St. Louis, MO, USA), and precipitated with ice-cold acetone (VWR, Radnor, PA, USA) overnight. Protein pellets were collected subsequently by centrifugation (9000× *g*, 4 °C), dissolved in 50 μL of 0.1 M triethyl ammonium bicarbonate (TEAB, Thermo Scientific, Rockford, IL, USA) and digested using 1 μL of trypsin (1 mg/mL, Promega; trypsin-to-protein ratio 1:35, at 37 °C overnight). The TMT 6 plex reagents (Thermo Scientific, Rockford, IL, USA) were prepared according to the manufacturer’s procedure. Then, reaction was quenched using 5% hydroxylamine (Sigma-Aldrich, St. Louis, MO, USA).

#### 2.3.2. Liquid Chromatography-Tandem Mass Spectrometry Analysis

TMT-labelled peptides were analysed by LC-MS/MS in high resolution using an Ultimate 3000 RSLCnano system (Dionex, Germering, Germany) coupled to a Q Exactive Plus mass spectrometer (Thermo Fisher Scientific, Bremen, Germany) as previously described [16].

The SEQUEST algorithm implemented in Proteome discoverer (version 2.3., Thermo Fisher Scientific) was used for protein identification and quantification from the Acquired MS/MS spectra. A database searching against Equus caballus FASTA files was made as previously described [9].

### 2.4. Statistics and Bioinformatics

Fold changes between groups were calculated as the log2(Mean(Group2)/Mean(Group1)). Due to the small number of individuals, normal distribution of values was not assumed; thus, the Mann–Whitney U test was used to assess the statistical significance of differentially expressed proteins between EGUS and control horses, and the Kruskal–Wallis H test was used to search for significant differences in protein expression between the three different groups (EGGD, ESGD, and control). For those proteins, Dunn’s multiple comparison test was used in a post hoc analysis to differentiate the specific variation between these groups. Statistical significance was considered when *p* < 0.05. All statistical analyses were implemented using Python3 and the SciPy [17] library.

Proteins were mapped to UniProt [18] entries and then annotated with their recommended gene names and descriptions. For functional characterization of the differentially expressed proteins, gene ontology (GO) enrichment analysis was performed with the ClueGo/CluePedia plugin for Cytoscape [19] and its functionalities to fuse and group functionally related terms to reduce redundancy.

## 3. Results

### 3.1. Proteomic Changes in the Saliva of Horses with Equine Gastric Ulcer Syndrome

The comparison between horses with EGUS and healthy controls is shown in Table 1. Twenty-five proteins showed upregulation in horses with EGUS, while four proteins were downregulated. The three proteins with the highest upregulation were transmembrane protease serine (TMPRSS11D), WD repeat Domain 1 (WDR1), and Serpin B5. The most downregulated were alpha-2-HS-glycoprotein (AHSG), jacalin-type lectin domain-containing protein (JAC) and 6-phosphogluconate dehydrogenase decarboxylating (PGD).

GO enrichment analysis showed that nine GO terms were upregulated in the saliva of horses with EGUS (Supplementary Figure S1). Among them, the most significant were the regulation of actin filament-based process and protein processing (Supplementary Table S1).

The comparison of horses with EGGD and healthy horses showed upregulation of 10 salivary proteins (Table 2), the most significant being transmembrane protease serine, protein S100-A9 (PSA-9), and peptidase S1 domain-containing protein, which according to our GO analysis were mostly associated with serine-type endopeptidase activity (Supplementary Figure S2 and Supplementary Table S2). No proteins were downregulated in horses with EGGD compared to healthy horses.

The comparison of the ESGD and healthy horses revealed 36 salivary proteins that were significantly differentially expressed (Table 3). From these, the proteins most upregulated in ESGD were serpin B5, WDR1, and glutaredoxin (GLRX) and transmembrane protease serine, of which the latter two showed changes of the same statistical significance. Two proteins were downregulated: deoxyribonuclease-1 (DNASE1) and PGD. The GO terms most related to ESGD were eicosanoid metabolic process, unsaturated fatty acid biosynthetic process, and protein processing (Supplementary Figure S3 and Supplementary Table S3).

Additionally, when comparing EGGD and ESGD groups, fourteen proteins were differentially expressed (Table 4). The most upregulated proteins in the ESGD group were arginase (ARG1), serpin B5, and the keratins 15 (KRT15) and 4 (KRT4). One protein was downregulated in ESGD compared to EGGD: the peptidase S1 domain-containing protein. The GO enrichment analysis between the significantly expressed proteins in the comparison of the EGGD and ESGD groups were characterized by nine different GO terms upregulated in the ESGD group compared to EGGD group (Supplementary Figure S4). The most significant were the adaptative immune response, T cell activation, and regulation of cell population proliferation (Supplementary Table S4).

### 3.2. Proteomic Changes in the Serum of Horses with Equine Gastric Ulcer Syndrome

The comparison between horses with EGUS and healthy horses is shown in Table 5. Five proteins were upregulated, while two were downregulated in horses with EGUS. The most upregulated proteins were fibrinogen alpha chain (FGA), complement factor I (CFI), and C3/C5 convertase (C2), and the only two downregulated proteins were complement component 8 subunit beta (C8B) and pregnancy zone protein. No GO terms were highlighted in the GO term enrichment analysis for the global comparison of horses with EGUS and controls.

Ten proteins were differentially abundant in the serum of horses with EGGD and healthy horses (Table 6). The three proteins that were most upregulated were FGA, C2, and hyaluronan binding protein 2 (HABP2). The most downregulated proteins were complement factor H (CFH), pregnancy zone protein, complement subcomponent C1r (C1R), and adiponectin A (C1QB). GO enrichment analysis revealed these downregulated proteins to be associated with the functional terms complement activation classical pathway and systemic lupus erythematosus (Supplementary Figure S5 and Supplementary Table S5).

In horses with ESGD, expression of nine proteins was significantly increased compared to healthy horses (Table 7). The most upregulated proteins were actin cytoplasmic 1 (ACTB), serpin family A member 6 (SERPINA6), and alpha-1-antiproteinase 2-like (SPI2). The most downregulated proteins were histidine-rich glycoprotein (HRG), C8B, and plasminogen (PLG). A total of six different GO terms were altered in this comparison (Supplementary Figure S6). The most significant upregulated GO terms were negative regulation of molecular function, serine-type endopeptidase inhibitor activity, and cellular protein metabolic process (Supplementary Table S6).

The comparison between the two EGUS groups showed eight proteins differently expressed, seven being upregulated and one downregulated in ESGD compared to EGGD (Table 8). The most relevant proteins with upregulated expression were C1r, ACTB, and EGF containing fibulin extracellular matrix protein 1, while only alpha-2-HS glycoprotein was found downregulated. GO terms analysis showed the enrichment of the postsynapse, synapse organization, and phagosome GO terms within these upregulated proteins (Supplementary Figure S7 and Supplementary Table S7).

## 4. Discussion

Our results provide preliminary evidence of EGUS-induced changes in the salivary protein profile, which could further differentiate between the two EGUS clinical forms, EGGD and ESGD. The number of differentially expressed proteins was more than three times higher in ESGD than in EGGD. Furthermore, the changes were not uniform when the horses with EGGD and ESGD were independently compared with the healthy horses. Therefore, it could be a better approach to assess the changes in saliva in EGGD and ESGD separately, instead of in all horses with EGUS.

Many of the proteins with the increased abundance of saliva in EGGD (e.g., TMPRSS11D, S100-A9, joining (J) chain, and adenosylhomocysteinase) share a common feature as they are involved in the regulation and activation of the immune system. This is in line with a recent report indicating the involvement of immune-mediated mechanisms and lymphoplasmacytic infiltration of the glandular mucosal inflammation in EGGD [5].

TMPRSS11D is a transmembrane serine protease and, as such, it is deeply involved in the inflammation and immune system reaction due to its ability to cleave the peptide bonds. Serine protease activation has been associated with an exacerbation of the immune response [20]. In addition, it has been reported in humans that Helicobacter pylori, a bacterium that could be involved in EGGD pathogenesis [1], can activate serine proteases, impairing the cellular repair and leading to connective tissue and extracellular matrix degradation [20]. However, to date, there remains conflict in the literature as to the role of bacteria in EGGD and there could be other factors that could activate serine proteases in this disease. For example, vascular and circulatory diseases can activate proteases [21]. This could potentially occur in EGGD, where circulatory disorders within the stomach leading to a reduced gastric glandular blood flow wall have been described as a possible cause [22].

S100-A9, also known as MRP14, is a Ca2 + binding protein belonging to the S100 family that forms a complex with S100-A8 called calprotectin. The upregulation of S100-A9 occurs in multiple immune system dysfunction diseases that result in excessive immune responses leading to autoimmune diseases and hypersensitivity reactions [23].

The joining (J) chain is a small polypeptide expressed by mucosal and glandular plasma cells, which regulates polymer formation of immunoglobulin (Ig) A and IgM. J chain shows a high affinity for the polymeric Ig receptor (pIgR) also known as transmembrane secretory component (SC). This epithelial glycoprotein mediates active external transfer of IgA and pentameric IgM to exocrine secretions. Therefore, it is a key protein in mucosal immunity [24].

The adenosylhomocysteinase, also named S-adenosylhomocysteine hydrolase, is an enzyme that converts S-adenosylhomocysteine into homocysteine and adenosine [25]. The in vivo activity of this enzyme depends on the function of another enzyme, adenosine deaminase (ADA). Both are located in the same chromosome and it seems that S-adenosylhomocysteine hydrolase, a eukaryotic enzyme, evolutionarily appeared later than ADA, which occurs in prokaryotes as well as eukaryotes [26,27]. Therefore, it could be postulated that the increase in adenosylhomocysteinase could also imply an increase in ADA, which is an enzyme related to immune function. This would be in line with the recent report on the increase in ADA in saliva in EGGD [28].

In the GO analysis of EGGD there was an activation of the pathway of the serine-type endopeptidase activity, which is in line with the increase found in the serine protease TMPRSS11D that is directly related to this GO term.

Saliva from horses diagnosed with ESGD showed a higher number of proteins with significant expression changes than the horses with EGGD. Some of those, such as serpin B5, WDR1, phosphoglycerate kinase 1 (PGK1), and keratins 15 and 4, have as a common feature the regulation of the growth of squamous epithelial cells, which represent the cytological substrate for ESGD [1].

Serpin B5 (also called maspin) is an intracellular serine protease inhibitor expressed in squamous epithelial cells, and increases in its expression have been described in ulcerative colitis in humans. This protein is involved in epithelial cell proliferation and resistance to apoptosis [29]. In addition, serpin B5 expression could be a marker of disease activity, since it was shown to be increased in over 90% of patients with active inflammatory bowel disease, being correlated to the activity of the disease [30]. This protein has been detected in saliva in humans associated to oral squamous cell carcinoma [31]. This could indicate that this protein is involved in alterations of the normal function, regulation, and metabolism of squamous cells that can occur in ESGD.

WDR1 is a protein that is involved in epithelial development and membrane epithelial cell junctions [32].

PGK1 has been found to increase in epithelial cells in the situation of anoxia and cellular damage and it has even been related to squamous cell neoplasm [33].

KRT15 and KRT8 are present in basal keratinocytes of all stratified epithelia, being also involved in epithelial cell proliferation [34]. In a previous study, other members of the keratin family, keratins 5 and 10, were found increased in the serum of horses with ESGD [3].

Besides serpin B5, WDR1, PGK1, and keratins 15 and 4, our results identify additional proteins involved in epithelial regulation that have a higher abundance in ESGD than in EGGD, thus implicating the presence of a complex disturbance in the squamous cell regulation. One of these proteins was arginase, which is involved in the growth of squamous cells. The upregulation of arginase activity has been described in gastric, breast, renal cell, and head and neck squamous cell carcinomas, thus making the inhibition of this enzyme a possible target for the treatment of these neoplasms [35]. On the other hand, arginase was lower in EGGD. Decreases in arginase activity in gastric ulcers have been reported in mice [36]. In addition to this, arginase has a regulatory role in gastrointestinal inflammation and low values could be related to an impaired tissue repair since upregulation of arginase increases the level of polyamines, which play a significant role in wound healing [37].

Overall, the changes in proteins in ESGD indicated an alteration of the squamous mucosa cells. This alteration has been found to be caused by acid and results in hyperkeratosis, erosions, and ulceration. Many of the risks for development of squamous disease relate to factors that allow or promote a more acidic gastric pH or increase exposure of the squamous mucosa to this acid [21].

In our study, the GO terms altered in horses with ESGD showed an activation of the pathways related to epithelium metabolism. Overall, when ESGD and EGGD were compared, the GO analysis in ESGD showed a tendency for activation of the pathways related to epithelium development and activation, while serine-type endopeptidase activity was more related to EGGD, as indicated above.

Serum of horses with ESGD and EGGD, showed a lower number of proteins with significant changes than saliva. Additionally, the GO analysis showed that the changes present in saliva mirror a higher number of upregulated processes. In addition, there was no substantial match between the pattern of proteome changes in the saliva and serum. This discrepancy in the number and types of protein changing between saliva and serum has also been observed in other diseases in different species, such as canine pyometra or mammary tumours [38,39], or in cows with mastitis [40]. Those results would indicate that changes in the composition of both fluids show a different response to the disease and can provide complementary information. These differences could be explained in part because there are analytes present in saliva that are locally produced in the salivary glands [41].

In a previous proteomic study analysing the saliva of horses with colic of intestinal aetiology and using the same technology, different proteins were identified compared to the current report [9]. These proteins were mostly related to a protective effect against inflammation and an impaired immune defence and antimicrobial capacity of the mucosa. This would reflect a different pathophysiological mechanism of this disease in comparison to ESGD or EGGD.

It is important to point out that this should be considered a pilot study. The sine qua non for the large-scale validation of these results should be the development of high-throughput assays for the quantitation of the main proteins that had different abundance levels between the groups. This large-scale validation should include a larger number of horses with ESGD and EGGD, as well as horses with other diseases, in order to determine the clinical sensitivity and specificity of these proteins, and to evaluate their possible use as biomarkers for diagnostic or monitoring purposes. In addition, since the gastroscopy exam of the horses with EGUS required a period of fasting, it would be interesting to investigate the possible effect of fasting in salivary protein variations.

## 5. Conclusions

In our study, horses with ESGD and EGGD showed different protein profiles in saliva when analysed by proteomics with a TMT-based approach. The most upregulated proteins in EGGD were related to immune activation whereas, in horses with ESGD, the proteins that changed were related to squamous cell regulation and growth. The proteins identified in our study, in addition to providing novel information about the pathophysiological mechanisms in these diseases, could have the potential to be new biomarkers for diagnosis or monitoring of EGGD and ESGD. Furthermore, our data provide additional evidence that EGGD and ESGD have two different pathophysiological mechanisms of disease and suggest that they should be considered two different diseases instead combining them into a single syndrome of EGUS.

## Figures and Tables

**Table 1:** Differentially abundant salivary proteins between horses with equine gastric ulcer syndrome (EGUS) (*n* = 12) and control group (*n* = 10). Columns indicate the accession number obtained from UniProt database, protein name, mean and SD abundances of control and EGUS group, Log2(FC) between EGUS and control groups, and *p*-value derived from Mann–Whitney analysis.

Accession (UniProt)	Protein Name	Mean and SD Control	Mean and SD EGUS	Log2(FC)	*p*-Value	Regulation in EGUS
F6QSG9	Transmembrane protease serine	0.63 ± 0.39	1.32 ± 0.79	1.04	0.003	UP
A0A3Q2I4F4	WD repeat domain 1	0.45 ± 0.27	0.79 ± 0.40	0.79	0.029	UP
F6YDZ0	Serpin B5	0.61 ± 0.39	1.06 ± 0.51	0.79	0.030	UP
F6W039	Rho GDP dissociation inhibitor alpha	0.59 ± 0.41	0.950.39	0.69	0.041	UP
P00559	Phosphoglycerate kinase 1	0.60 ± 0.38	0.94 ± 0.42	0.65	0.041	UP
F6Q818	Arachidonate 15-lipoxygenase type B	0.66 ± 0.35	1.02 ± 0.41	0.62	0.019	UP
F6SG30	Protein S100-A9	1.03 ± 0.38	1.560.46	0.60	0.006	UP
F7DMA1	Carboxypeptidase M	0.69 ± 0.32	1.02 ± 0.29	0.57	0.013	UP
F6YA47	Alpha-2-macroglobulin like 1	0.88 ± 0.40	1.31 ± 0.36	0.57	0.018	UP
A0A0B4J1C4	Joining chain of multimeric IgA and IgM	0.82 ± 0.16	1.20 ± 0.51	0.54	0.021	UP
F6T8J7	LY6/PLAUR domain containing 3	0.90 ± 0.25	1.31 ± 0.31	0.54	0.004	UP
F6WR95	Sulfhydryl oxidase	0.91 ± 0.20	1.29 ± 0.19	0.51	0.001	UP
F6SP02	14-3-3 protein theta	0.85 ± 0.34	1.21 ± 0.43	0.50	0.047	UP
A0A5F5PSG4	Desmoglein 2	0.95 ± 0.34	1.30 ± 0.36	0.46	0.047	UP
A0A3Q2KJH8	Desmocollin 2	1.03 ± 0.37	1.42 ± 0.36	0.45	0.023	UP
F6S6J4	Peroxiredoxin-1	0.88 ± 0.48	1.19 ± 0.35	0.43	0.041	UP
A0A5F5PRI0	Nucleoside diphosphate kinase	0.86 ± 0.42	1.14 ± 0.44	0.40	0.041	UP
Q28372	Gelsolin	0.79 ± 0.30	1.04 ± 0.27	0.39	0.018	UP
F7DW69	Heat shock 70 kDa protein 1A	0.94 ± 0.31	1.23 ± 0.36	0.38	0.041	UP
A0A3Q2GYE5	Kallikrein related peptidase 12	0.85 ± 0.31	1.09 ± 0.34	0.36	0.041	UP
A0A3Q2LCR2	Glucose-6-phosphate isomerase	0.88 ± 0.24	1.13 ± 0.28	0.36	0.047	UP
A0A5S7NAP8	Chloride channel accessory 2	0.97 ± 0.24	1.23 ± 0.30	0.35	0.035	UP
A0A3Q2HWQ6	C3-beta-c	0.89 ± 0.32	1.10 ± 0.27	0.30	0.041	UP
F6PH38	Fibrinogen beta chain	0.87 ± 0.21	1.07 ± 0.26	0.30	0.041	UP
A0A5F5PF02	Tropomyosin alpha-4 chain	0.93 ± 0.25	1.14 ± 0.23	0.29	0.041	UP
Q4AEE3	Deoxyribonuclease-1	0.98 ± 0.26	0.79 ± 0.28	−0.31	0.035	DOWN
F7C450	Alpha-2-HS-glycoprotein	1.33 ± 0.60	0.91 ± 0.35	−0.54	0.041	DOWN
A0A3Q2I8J7	Jacalin-type lectin domain-containing protein	1.19 ± 0.45	0.79 ± 0.36	−0.58	0.030	DOWN
F7D917	6-phosphogluconate dehydrogenase decarboxylating	1.12 ± 0.75	0.55 ± 0.25	−1.03	0.007	DOWN

SD: standard deviation; EGUS: equine gastric ulcer syndrome; FC: fold change.

**Table 2:** Differentially abundant salivary proteins between horses with equine glandular gastric disease (EGGD) (*n* = 6) and control group (*n* = 10). Columns indicate the accession number obtained from UniProt database, protein name, mean and SD abundances of control and EGGD groups, Log2(FC) between EGGD and control groups, and *p*-value derived from Kruskal–Wallis analysis.

Accession (UniProt)	Protein Name	Mean and SD Control	Mean and SD EGGD	Log2(FC)	*p*-Value	Regulation in EGGD
F6QSG9	Transmembrane protease serine	0.63 ± 0.39	1.49 ± 1.04	1.23	0.025	UP
F6SG30	Protein S100-A9	1.03 ± 0.38	1.66 ± 0.49	0.69	0.01	UP
A0A3Q2HN65	Peptidase S1 domain-containing protein	1.08 ± 0.37	1.70 ± 0.60	0.64	0.019	UP
F6Q818	Arachidonate 15-lipoxygenase type B	0.65 ± 0.35	0.99 ± 0.38	0.59	0.043	UP
F6YA47	Alpha-2-macroglobulin like 1	0.88 ± 0.41	1.30 ± 0.40	0.56	0.043	UP
A0A0B4J1C4	Joining chain of multimeric IgA and IgM	0.82 ± 0.16	1.16 ± 0.29	0.50	0.019	UP
F6T8J7	LY6/PLAUR domain containing 3	0.90 ± 0.25	1.26 ± 0.34	0.48	0.019	UP
F6 × 058	Transmembrane protease serine	0.89 ± 0.31	1.24 ± 0.29	0.47	0.033	UP
F6WR95	Sulfhydryl oxidase	0.91 ± 0.20	1.24 ± 0.21	0.44	0.007	UP
A0A5F5PFG3	Adenosylhomocysteinase	1.08 ± 0.22	1.39 ± 0.24	0.29	0.049	UP

SD: standard deviation; EGGD: equine glandular gastric disease; FC: fold change.

**Table 3:** Differentially abundant salivary proteins between horses with equine squamous gastric disease (ESGD) (*n* = 6) and control group (*n* = 10). Columns indicate the accession number obtained from UniProt database, protein name, mean and SD abundances of control and ESGD groups, Log2(FC) between ESGD and control groups, and *p*-value derived from Kruskal-Wallis analysis.

Accession (UniProt)	Protein Name	Mean and SD Control	Mean and SD ESGD	Log2(FC)	*p*-Value	Regulation in ESGD
F6YDZ0	Serpin B5	0.61 ± 0–39	1.37 ± 0.51	1.16	0.011	UP
A0A3Q2I4F4	WD repeat domain 1	0.46 ± 0.27	0.92 ± 0.47	1.01	0.025	UP
F6QR81	Glutaredoxin	0.95 ± 0.35	1.71 ± 1.07	0.85	0.044	UP
F6QSG9	Transmembrane protease serine	0.64 ± 0.39	1.14 ± 0.34	0.84	0.008	UP
P00559	Phosphoglycerate kinase 1	0.60 ± 0.38	1.07 ± 0.45	0.83	0.033	UP
F6 × 058	Transmembrane protease serine	0.90 ± 0.31	1.54 ± 0.41	0.78	0.004	UP
F7CJ82	Arginase	0.98 ± 0.52	1.60 ± 0.91	0.70	0.044	UP
F6Q1M4	Keratin 15	0.63 ± 0.22	1.01 ± 0.29	0.69	0.020	UP
A0A5F5PRI0	Nucleoside diphosphate kinase	0.86 ± 0.42	1.38 ± 0.45	0.68	0.020	UP
Q8HZM6	Annexin A1	0.84 ± 0.36	1.34 ± 0.43	0.68	0.034	UP
F7D5 × 8	Keratin 4	0.91 ± 0.43	1.44 ± 0.56	0.67	0.034	UP
F7DMA1	Carboxypeptidase M	0.69 ± 0.32	1.09 ± 0.28	0.66	0.019	UP
F6Q818	Arachidonate 15-lipoxygenase type B	0.66 ± 0.35	1.04 ± 0.44	0.65	0.049	UP
F6SP02	14-3-3 protein theta	0.85 ± 0.34	1.34 ± 0.43	0.65	0.034	UP
Q28372	Gelsolin	0.79 ± 0.30	1.19 ± 0.27	0.60	0.011	UP
F6T8J7	LY6/PLAUR domain containing 3	0.90 ± 0.25	1.36 ± 0.26	0.59	0.011	UP
A0A3Q2KJH8	Desmocollin 2	1.03 ± 0.37	1.55 ± 0.42	0.58	0.020	UP
A0A3Q2GYE5	Kallikrein related peptidase 12	0.85 ± 0.31	1.27 ± 0.34	0.58	0.026	UP
F6YA47	Alpha-2-macroglobulin like 1	0.88 ± 0.40	1.31 ± 0.32	0.57	0.044	UP
A0A3Q2HC63	Calmodulin like 5	0.97 ± 0.31	1.44 ± 0.44	0.57	0.026	UP
F6S6J4	Peroxiredoxin-1	0.88 ± 0.48	1.31 ± 0.27	0.57	0.034	UP
F6WR95	Sulfhydryl oxidase	0.91 ± 0.20	1.35 ± 0.17	0.57	0.002	UP
F6RGN2	Fatty acid binding protein 5	1.05 ± 0.47	1.55 ± 0.40	0.56	0.026	UP
F6TZS9	Triosephosphate isomerase	0.72 ± 0.33	1.02 ± 0.36	0.51	0.029	UP
F6SG30	Protein S100-A9	1.03 ± 0.38	1.46 ± 0.40	0.50	0.034	UP
F6SX07	Galectin	1.12 ± 0.69	1.58 ± 0.42	0.49	0.044	UP
A0A3Q2HWQ6	C3-beta-c	0.89 ± 0.32	1.25 ± 0.25	0.49	0.011	UP
F7DW69	Heat shock 70 kDa protein 1A	0.94 ± 0.31	1.31 ± 0.27	0.47	0.026	UP
F6PH38	Fibrinogen beta chain	0.87 ± 0.21	1.20 ± 0.22	0.47	0.011	UP
A0A3Q2HT63	Serpin B3	0.94 ± 0.35	1.26 ± 0.31	0.43	0.034	UP
F6VJR6	Alpha-1B-glycoprotein	0.82 ± 0.22	1.05 ± 0.16	0.36	0.034	UP
B7XH73	L-lactate dehydrogenase	0.91 ± 0.48	1.16 ± 0.18	0.35	0.044	UP
F7BPX8	Cathepsin L1	0.96 ± 0.35	1.20 ± 0.22	0.33	0.011	UP
A0A5F5PF02	Tropomyosin alpha-4 chain	0.93 ± 0.25	1.17 ± 0.20	0.32	0.044	UP
Q4AEE3	Deoxyribonuclease-1	0.98 ± 0.26	0.74 ± 0.18	−0.41	0.044	DOWN
F7D917	6-phosphogluconate dehydrogenase decarboxylating	1.12 ± 0.75	0.46 ± 0.10	−1.27	0.008	DOWN

SD: standard deviation; ESGD: equine squamous gastric disease; FC: fold change.

**Table 4:** Differentially abundant salivary proteins between horses with equine glandular gastric disease (*n* = 6) and equine squamous gastric disease (*n* = 6). Columns indicate the accession number obtained from UniProt database, protein name, mean and SD abundances of EGGD and ESGD groups, Log2(FC) between ESGD and EGGD groups, and *p*-value derived from Kruskal–Wallis analysis.

Accession (UniProt)	Protein Name	Mean and SD EGGD	Mean and SD ESGD	Log2(FC)	*p*-Value	Regulation in ESGD
F7CJ82	Arginase	0.80 ± 0.46	1.60 ± 0.91	1.00	0.023	UP
F6YDZ0	Serpin B5	0.75 ± 0.28	1.37 ± 0.51	0.88	0.023	UP
F6Q1M4	Keratin 15	0.55 ± 0.07	1.01 ± 0.29	0.88	0.003	UP
F7D5 × 8	Keratin 4	0.81 ± 0.18	1.44 ± 0.56	0.83	0.023	UP
A0A5F5PRI0	Nucleoside diphosphate kinase	0.89 ± 0.24	1.38 ± 0.45	0.64	0.015	UP
A0A3Q2LHM4	SEC14-like protein 4	0.84 ± 0.17	1.23 ± 0.25	0.55	0.015	UP
F6RGN2	Fatty acid binding protein 5	1.07 ± 0.33	1.55 ± 0.40	0.53	0.015	UP
Q8HZM6	Annexin A1	0.95 ± 0.36	1.34 ± 0.43	0.50	0.046	UP
A0A3Q2GYE5	Kallikrein related peptidase 12	0.92 ± 0.22	1.27 ± 0.34	0.47	0.046	UP
Q28372	Gelsolin	0.88 ± 0.17	1.19 ± 0.24	0.44	0.033	UP
A0A3Q2HWQ6	C3-beta-c	0.95 ± 0.21	1.25 ± 0.25	0.41	0.015	UP
F7BPX8	Cathepsin L1	0.92 ± 0.11	1.20 ± 0.22	0.39	0.010	UP
A0A3Q2GTU4	Fructose-bisphosphate aldolase	1.05 ± 0.20	1.35 ± 0.12	0.37	0.033	UP
A0A3Q2HN65	Peptidase S1 domain-containing protein	1.70 ± 0.60	1.30 ± 0.71	−0.39	0.046	DOWN

SD: standard deviation; EGGD: equine glandular gastric disease; ESGD: equine squamous gastric disease; FC: fold change.

**Table 5:** Differentially abundant serum proteins between horses with equine gastric ulcer syndrome (EGUS) (*n* = 12) and controls (*n* = 10). Columns indicate the accession number obtained from UniProt database, protein name, mean and SD abundances of control and EGUS groups, Log2(FC) between EGUS and control groups, and *p*-value derived from Mann–Whitney analysis.

Accession (UniProt)	Protein Name	Mean and SD Control	Mean and SD EGUS	Log2(FC)	*p*-Value	Regulation in EGUS
A0A3Q2HTG2	Fibrinogen alpha chain	0.90 ± 0.08	1.20 ± 0.54	0.42	0.022	UP
F7APU2	Complement factor I	0.98 ± 0.07	1.13 ± 0.30	0.20	0.049	UP
F6PPQ0	C3/C5 convertase	0.94 ± 0.07	1.08 ± 0.06	0.19	0.006	UP
F7DRS2	Serpin family A member 6	0.97 ± 0.08	1.08 ± 0.11	0.15	0.024	UP
A0A3Q2I8Y6	Alpha-2-glycoprotein 1 zinc-binding	0.99 ± 0.06	1.05 ± 0.07	0.08	0.036	UP
A0A3Q2LBD1	Complement component 8 subunit beta	1.01 ± 0.05	0.91 ± 0.06	−0.14	0.000	DOWN
F6REX3	Pregnancy zone protein	1.11 ± 0.36	0.85 ± 0.23	−0.39	0.042	DOWN

SD: standard deviation; EGUS: equine gastric ulcer syndrome; FC: fold change.

**Table 6:** Differentially abundant serum proteins between horses with equine glandular gastric disease (*n* = 6) and controls (*n* = 10). Columns indicate the accession number obtained from UniProt database, protein name, mean and SD abundances of control and EGGD groups, Log2(FC) between EGGD and control groups, and *p*-value derived from Kruskal–Wallis analysis.

Accession (UniProt)	Protein Name	Mean Control	Mean EGGD	Log2(FC)	*p*-Value	Regulation in EGGD
A0A3Q2HTG2	Fibrinogen alpha chain	1.09 ± 0.08	0.90 ± 0.23	0.29	0.029	UP
F6PPQ0	C3/C5 convertase	1.08 ± 0.07	0.94 ± 0.07	0.19	0.005	UP
F6VZ73	Hyaluronan binding protein 2	1.05 ± 0.09	0.95 ± 0.10	0.14	0.046	UP
F6PKE1	Inhibitor of carbonic anhydrase	1.05 ± 0.05	0.98 ± 0.06	0.09	0.041	UP
F6ZR63	Complement factor H	0.87 ± 0.04	0.95 ± 0.06	−0.11	0.029	DOWN
A0A3Q2LBD1	Complement component 8 subunit beta	0.93 ± 0.05	1.01 ± 0.03	−0.12	0.004	DOWN
Q95M34	Immunoglobulin gamma 1 heavy chain constant region (Fragment)	0.87 ± 0.10	0.99 ± 0.07	−0.19	0.013	DOWN
F7BUV8	Adiponectin A	0.96 ± 0.17	1.12 ± 0.08	−0.22	0.046	DOWN
F6Z5L1	Complement subcomponent C1r	0.69 ± 0.32	0.92 ± 0.31	−0.43	0.037	DOWN
F6REX3	Pregnancy zone protein	0.82 ± 0.36	1.11 ± 0.16	−0.44	0.046	DOWN

SD: standard deviation; EGGD: equine glandular gastric disease; FC: fold change.

**Table 7:** Differentially abundant serum proteins between horses with equine squamous gastric disease (ESGD) (*n* = 6) and controls (*n* = 10). Columns indicate the accession number obtained from UniProt database, protein name, mean and SD abundances of control and ESGD groups, Log2(FC) between ESGD and control groups, and *p*-value derived from Kruskal–Wallis analysis.

Accession (UniProt)	Protein Name	Mean Control	Mean ESGD	Log2(FC)	*p*-Value	Regulation in EGSD
A0A3Q2KTQ9	Actin cytoplasmic 1	1.14 ± 0.21	0.94 ± 0.19	0.28	0.049	UP
F7DRS2	Serpin family A member 6	1.10 ± 0.08	0.97 ± 0.11	0.17	0.029	UP
F7CSL8	Alpha-1-antiproteinase 2-like	1.13 ± 0.08	1.00 ± 0.12	0.17	0.049	UP
F7CZW9	Serpin family G member 1	1.03 ± 0.09	0.93 ± 0.05	0.15	0.049	UP
A0A3Q2I8Y6	Alpha-2-glycoprotein 1 zinc-binding	1.06 ± 0.06	0.99 ± 0.06	0.10	0.049	UP
F7BM31	Serpin family D member 1	0.92 ± 0.06	0.99 ± 0.03	−0.10	0.025	DOWN
F6USP9	Plasminogen	0.92 ± 0.05	1.02 ± 0.07	−0.14	0.029	DOWN
A0A3Q2LBD1	Complement component 8 subunit beta	0.89 ± 0.05	1.01 ± 0.07	−0.18	0.003	DOWN
F6ZI35	Histidine rich glycoprotein	0.89 ± 0.13	1.00 ± 0.10	−0.18	0.049	DOWN

SD: standard deviation; ESGD: equine squamous gastric disease; FC: fold change.

**Table 8:** Differentially abundant serum proteins between horses with equine squamous gastric disease (ESGD) (*n* = 6) and equine glandular gastric disease (EGGD) (*n* = 6). Columns indicate the accession number obtained from UniProt database, protein name, mean and SD abundances of EGGD and ESGD groups, Log2(FC) between ESGD and EGGD groups, and *p*-value derived from Kruskal–Wallis analysis.

Accession (UniProt)	Protein Name	Mean EGGD	Mean ESGD	Log2(FC)	*p*-Value	Regulation in EGSD
F6Z5L1	Complement subcomponent C1r	0.68 ± 0.29	0.93 ± 0.31	0.44	0.041	UP
A0A3Q2KTQ9	Actin cytoplasmic 1	0.87 ± 0.11	1.14 ± 0.19	0.38	0.041	UP
F6PVG3	EGF containing fibulin extracellular matrix protein 1	0.92 ± 0.11	1.14 ± 0.16	0.30	0.041	UP
F7CSL8	Alpha-1-antiproteinase 2-like	0.94 ± 0.15	1.12 ± 0.12	0.24	0.041	UP
Q95M34	Immunoglobulin gamma 1 heavy chain constant region (Fragment)	0.86 ± 0.07	0.99 ± 0.08	0.19	0.028	UP
F7BUV8	Adiponectin A	0.96 ± 0.08	1.06 ± 0.08	0.15	0.041	UP
F6ZR63	Complement factor H	0.87 ± 0.06	0.95 ± 0.04	0.11	0.028	UP
F7C450	Alpha-2-HS-glycoprotein	0.95 ± 0.11	0.85 ± 0.08	−0.16	0.040	DOWN

SD: standard deviation; EGGD: equine glandular gastric disease; ESGD: equine squamous gastric disease; FC: fold change.

## Data Availability

Not applicable.

## Supplementary Materials

The following supporting information can be downloaded at: www.mdpi.com/article/10.3390/ani12091169/s1, Figure S1: Interactome of GO terms differentially expressed in the saliva of horses with EGUS, and their intermediate proteins. Figure S2: Interactome of GO terms differentially expressed in the saliva of horses with EGGD compared with controls, and their intermediate proteins. Figure S3: Interactome of GO terms differentially expressed in the saliva of horses with ESGD compared with controls, and their intermediate proteins. Figure S4: Interactome of GO terms differentially expressed in the saliva of horses with ESGD compared with horses with EGGD, and their intermediate proteins. Figure S5: Interactome of GO terms differentially expressed in the serum of horses with EGGD compared with controls, and their intermediate proteins. Figure S6: Interactome of GO terms differentially expressed in the serum of horses with ESGD compared with controls, and their intermediate proteins. Figure S7: Interactome of GO terms differentially expressed in the serum of horses with ESGD compared with horses with EGGD, and their intermediate proteins. Table S1: Groups of most representative GO terms in the saliva of horses with EGUS compared with controls. Table S2: Groups of most representative GO terms in the saliva of horses with EGGD compared with controls. Table S3: Groups of most representative GO terms in the saliva of horses with ESGD compared with controls. Table S4: Groups of most representative GO terms in the saliva of horses with ESGD compared with EGGD. Table S5: Groups of most representative GO terms in the serum of horses with EGGD compared with controls. Table S6: Groups of most representative GO terms in the serum of horses with ESGD compared with controls. Table S7: Groups of most representative GO terms in the serum of horses with ESGD compared with EGGD.
