# Supplementary material for: Changes in Proteins in Saliva and Serum in Equine Gastric Ulcer Syndrome Using a Proteomic Approach
Source: Animals (Basel). 2022 May 2;12(9):1169. doi: 10.3390/ani12091169 (PMC9103582; doi:10.3390/ani12091169)
Supplement: Supplementary file 1 [file animals-12-01169-s001.zip › Supplementary Figures.pdf]

Supplementary data.

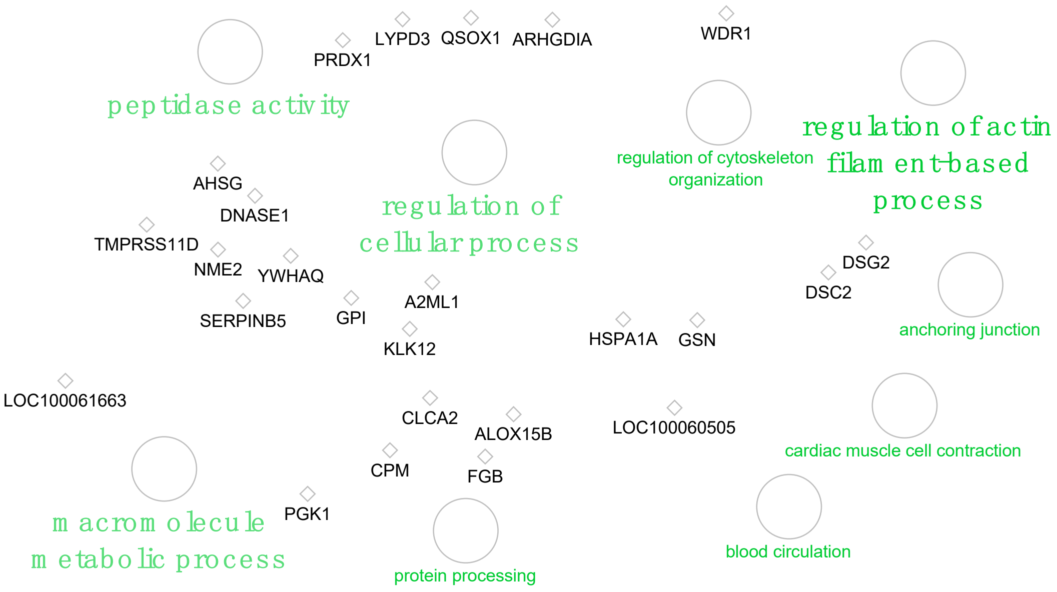

Supplementary Figure S1. Interactome of GO terms differentially expressed in the saliva of horses with EGUS, and their intermediate proteins. These analyses have been done with the Cytoscape application ClueGO and the REVIGO tool for GO terms selection. GO terms and proteins over-expressed in EGUS are in green, lower-expressed are in red. GO terms in grey could not be attributed specifically to over or lower expressed terms/proteins. GO terms in bold represent GO terms selected to be the most representative of their GO group defined by the REVIGO tool.

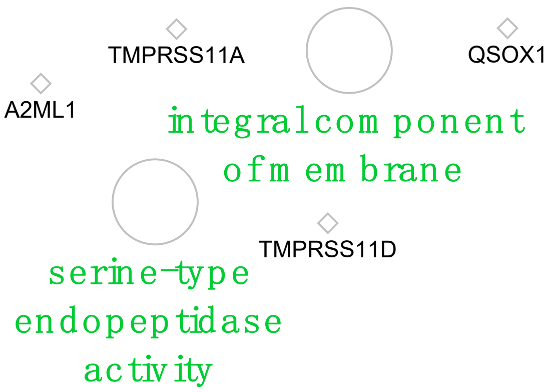

Supplementary Figure S2. Interactome of GO terms differentially expressed in the saliva of horses with EGGD compared with controls, and their intermediate proteins. These analyses have been done with the Cytoscape application ClueGO and the REVIGO tool for GO terms selection. GO terms and proteins over-expressed in EGGD are in green, lower-expressed are in red. GO terms in grey could not be attributed specifically to over or lower expressed terms/proteins. GO terms in bold represent GO terms selected to be the most representative of their GO group defined by the REVIGO tool.

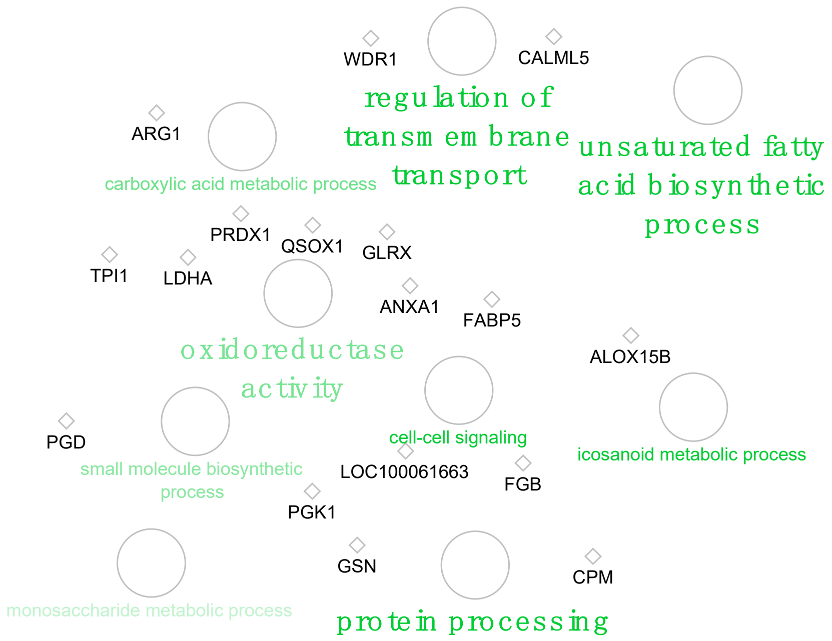

Supplementary Figure S3. Interactome of GO terms differentially expressed in the saliva of horses with ESGD compared with controls, and their intermediate proteins. These analyses have been done with the Cytoscape application ClueGO and the REVIGO tool for GO terms selection. GO terms and proteins over-expressed in ESGD are in green, lower-expressed are in red. GO terms in grey could not be attributed specifically to over or lower expressed terms/proteins. GO terms in bold represent GO terms selected to be the most representative of their GO group defined by the REVIGO tool.

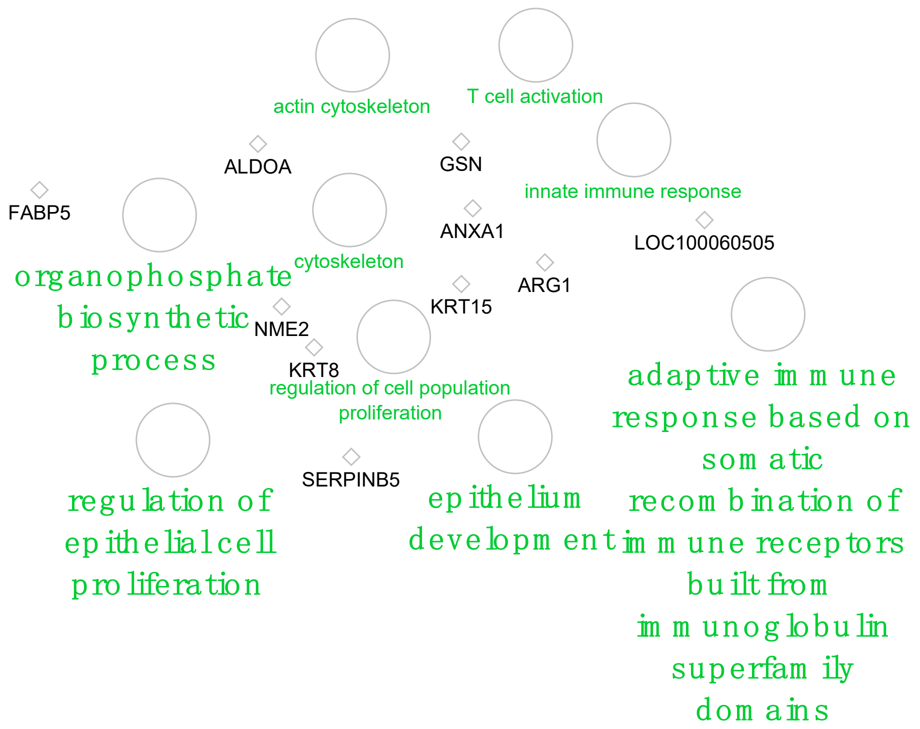

Supplementary Figure S4. Interactome of GO terms differentially expressed in the saliva of horses with ESGD compared with horses with EGGD, and their intermediate proteins. These analyses have been done with the Cytoscape application ClueGO and the REVIGO tool for GO terms selection. GO terms and proteins over-expressed in ESGD are in green, lower-expressed are in red. GO terms in grey could not be attributed specifically to over or lower expressed terms/proteins. GO terms in bold represent GO terms selected to be the most representative of their GO group defined by the REVIGO tool.

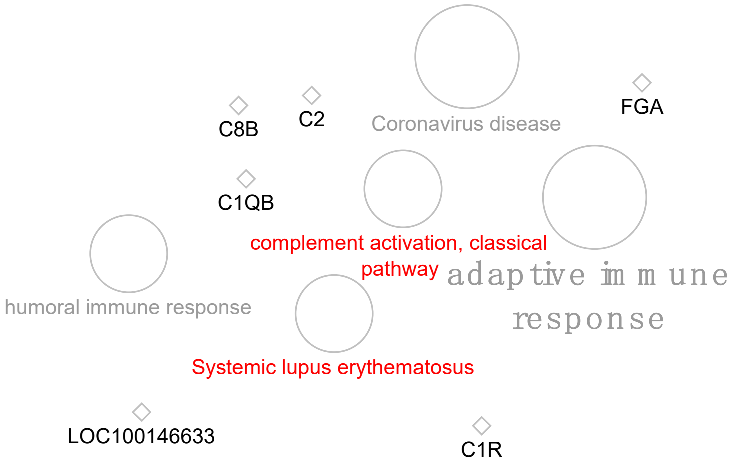

Supplementary Figure S5. Interactome of GO terms differentially expressed in the serum of horses with EGGD compared with controls, and their intermediate proteins. These analyses have been done with the Cytoscape application ClueGO and the REVIGO tool for GO terms selection. GO terms and proteins over-expressed in EGGD are in green, lower-expressed are in red. GO terms in grey could not be attributed specifically to over or lower expressed terms/proteins. GO terms in bold represent GO terms selected to be the most representative of their GO group defined by the REVIGO tool.

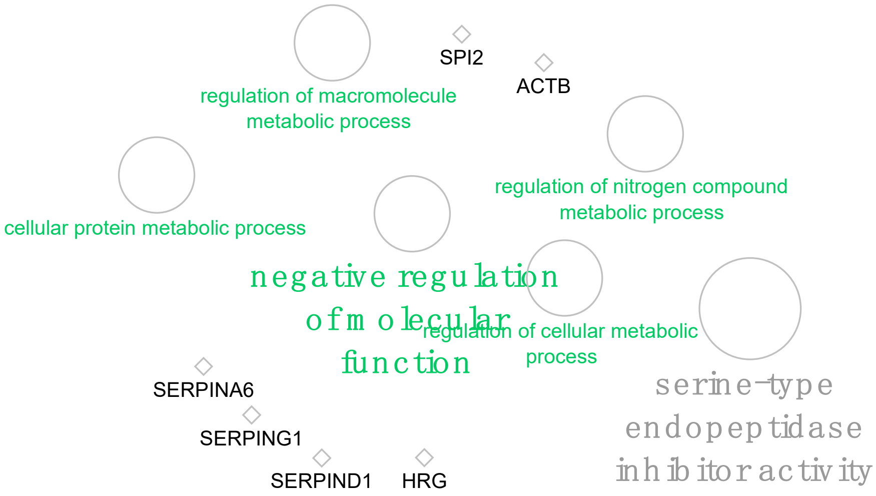

Supplementary Figure S6. Interactome of GO terms differentially expressed in the serum of horses with ESGD compared with controls, and their intermediate proteins. These analyses have been done with the Cytoscape application ClueGO and the REVIGO tool for GO terms selection. GO terms and proteins over-expressed in ESGD are in green, lower-expressed are in red. GO terms in grey could not be attributed specifically to over or lower expressed terms/proteins. GO terms in bold represent GO terms selected to be the most representative of their GO group defined by the REVIGO tool.

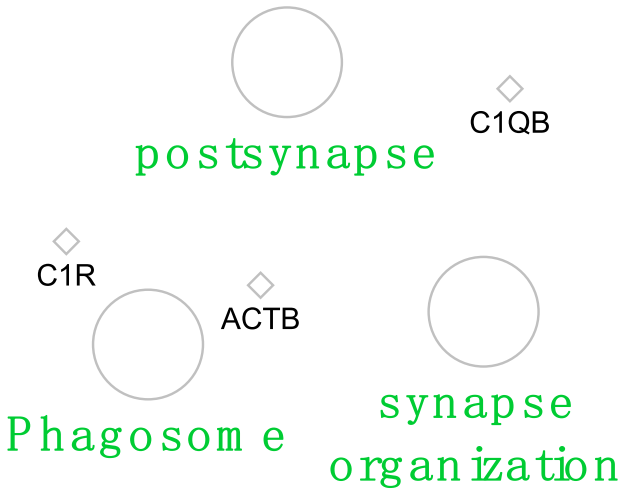

Supplementary Figure S7. Interactome of GO terms differentially expressed in the serum of horses with ESGD compared with horses with EGGD, and their intermediate proteins. These analyses have been done with the Cytoscape application ClueGO and the REVIGO tool for GO terms selection. GO terms and proteins over-expressed in ESGD are in green, lower-expressed are in red. GO terms in grey could not be attributed specifically to over or lower expressed terms/proteins. GO terms in bold represent GO terms selected to be the most representative of their GO group defined by the REVIGO tool
